# Altered association of plasmatic oxytocin with affective stress response in alcohol use disorder

**DOI:** 10.1038/s41398-025-03522-0

**Published:** 2025-10-20

**Authors:** Annalina V. Mayer, Yana Schwarze, Janine Stierand, Johanna Voges, Alexander Schröder, Janina von der Gablentz, Klaus Junghanns, Oliver Voß, Sören Krach, Frieder M. Paulus, Lena Rademacher

**Affiliations:** 1 Social Neuroscience Lab, Department of Psychiatry and Psychotherapy, University of Lübeck, Lübeck, Germany; 2 Center of Brain, Behavior and Metabolism (CBBM), University of Lübeck, Lübeck, Germany; 3 Department of Psychiatry and Psychotherapy, University of Lübeck, Lübeck, Schleswig-Holstein Germany; 4 Department of Psychosomatic Medicine, Charité - Universitätsmedizin Berlin, Corporate Member of Freie Universität Berlin, Humboldt-Universität zu Berlin, and Berlin Institute of Health, Berlin, Germany; 5 AMEOS Clinic Lübeck, Department of Substance Use Disorders, Lübeck, Germany; 6 Clinic for Addictive Behaviour and Addiction Medicine, Klinikum Stuttgart, Stuttgart, Germany

**Keywords:** Addiction, Human behaviour

## Abstract

Oxytocin has been proposed to play a role in the development and maintenance of alcohol use disorder (AUD) through its interactions with stress pathways. Empirical evidence that indicates altered associations between endogenous oxytocin and stress reactivity in AUD is currently lacking. In this study, we investigated baseline plasmatic oxytocin concentrations of early-abstinent patients with AUD (*N* = 40) and matched healthy control participants (*N* = 37), who completed the Trier Social Stress Test (TSST) as well as a control task on two separate visits. We measured salivary cortisol and pulse rate as indicators of a physiological stress response, and anxiety ratings as an indicator of an affective stress response at multiple time points. Baseline oxytocin levels did not significantly differ between the groups. However, our results suggest an altered association of oxytocin and affective stress responses in participants with AUD: while participants with AUD showed a positive relationship between plasmatic oxytocin levels and stress-induced anxiety increase, the opposite relationship was found in control participants. We did not find evidence for an association of oxytocin with physiological stress responses. The present findings suggest that oxytocin may not possess the same anxiolytic properties in individuals with AUD under stress conditions as it does in healthy subjects. This is an important consideration for future hypotheses on the use of exogenous oxytocin in the treatment of AUD.

## Introduction

Various studies have highlighted the importance of stress in the development and maintenance of alcohol use disorder (AUD) [1]. The relationship between heavy alcohol consumption and stress seems to be reciprocal: while repeated exposure to stressful situations is an important risk factor for AUD and increases the risk of relapse [2, 3], chronic alcohol misuse itself stimulates the release of major stress hormones [4]. In the long run, the body’s chronic efforts to adapt to repeated or prolonged stress lead to a cumulative physiological toll on the individual [5]. In line with this, attenuated physiological stress responses have been observed in individuals with AUD, suggesting a dysregulation of the hypothalamic–pituitary–adrenal (HPA)-axis and the autonomic nervous system after prolonged periods of heavy alcohol use [6–12]. Notably, while physiological stress responses are blunted, affective stress responses such as feelings of anxiety, worry and unease seem to be comparable or even more pronounced in individuals with AUD relative to healthy individuals, even in long-term abstinence [12–14].

Recently, the hormone and neuropeptide oxytocin has become a focus of research on the link between stress and addiction. A large body of research has discussed the role of oxytocin in modulating stress responses [15, 16]. For example, results from rodent studies show that inhibiting the action of endogenous oxytocin in the brain through administration of an oxytocin receptor antagonist results in increased basal and stress-induced activity of the HPA-axis [17, 18]. In humans, converging evidence suggests that plasmatic and salivary oxytocin levels rapidly increase during and after stressful conditions [19–22]. This short-term increase is thought to dampen physiological stress responses by modulating HPA-axis activity [23]. Accordingly, higher overall plasma oxytocin levels were associated with dampened physiological responses to different stressors in postpartum mothers [24]. Findings on the relationship of oxytocin and affective stress responses, however, have been mixed [25]. Although some studies suggest that oxytocin may facilitate acute fear responses, most studies report an anxiolytic effect of oxytocin in healthy individuals [26], suggesting lower levels of anxiety in those with higher endogenous oxytocin levels [27–29] and after intranasal oxytocin administration [30, 31].

Aside from its involvement in stress regulation, oxytocin has also been suggested to modulate alcohol-related responses, such as craving [32, 33], tolerance [34], and withdrawal symptoms [35], although other studies could not find an effect [36, 37]. It has been proposed that alcohol and other substance use disorders are characterized by alterations in the oxytocin system [38, 39]. This is supported by evidence from animal models of AUD [40, 41], human studies linking alcohol consumption to plasma oxytocin levels and DNA methylation in the oxytocin receptor gene [42], as well as clinical studies showing elevated plasma oxytocin levels in patients with AUD during the first days of abstinence compared to healthy individuals [43, 44].

It has been proposed that oxytocin may exert its effects on addiction by interacting with stress pathways [45]. For example, central administration of oxytocin or the oxytocin analog carbetocin was found to inhibit drug-seeking behavior after stress in rodents [46, 47]. In humans, intranasal oxytocin was similarly found to decrease stress-induced anxiety and craving of cannabis in a randomized controlled trial [48]. These findings suggest that by dampening stress responses oxytocin may facilitate abstinence [45]. This makes the oxytocin system a potential pharmacological target for treatments of substance use disorders [49]. However, although preclinical studies on the effects of exogenous oxytocin appear promising, no research has demonstrated a direct connection between altered endogenous oxytocin levels and stress reactivity in individuals with substance use disorders, including AUD. In addition, research in other clinical groups has shown that the effects of oxytocin can also be anxiogenic rather than anxiolytic. For example, in individuals with depression, oxytocin led to increased anxiety levels over the course of a psychotherapy session [50]. Furthermore, individuals who reported higher levels of social isolation were shown to be less responsive to the effects of oxytocin on cardiovascular responsivity [51]. One possible explanation for these effects could be that oxytocin modulates interoceptive signals and self-referential processing, which may result in different outcomes depending on person-dependent variables such as prior experiences [52].

The aim of the present study was to examine associations between plasmatic oxytocin levels with affective and physiological responses to psychosocial stress. To test this, participants with and without AUD underwent the Trier Social Stress Test (TSST), a validated task that reliably induces stress, as well as a control task lacking the elements of stressful social evaluation. Salivary cortisol samples, pulse rate, and affect ratings were collected before, during and after the tasks and their association with baseline plasmatic oxytocin levels was examined. For healthy control participants, based on research showing anxiolytic effects of oxytocin, we expected lower stress responses in individuals with higher baseline oxytocin levels. Given the previous findings in other clinical groups, we expected that there is a less pronounced anxiolytic effect in participants with AUD or even the reverse, an anxiogenic effect. Thus, we examined interaction effects of group, task and oxytocin on physiological and affective stress measures.

## Methods

### Participants

The sample consisted of 42 participants with a DSM-5 diagnosis of moderate or severe alcohol use disorder (fulfilling 5–11 criteria) and 38 healthy control participants between the ages of 24 and 59 years. Two participants with AUD and one control participant discontinued the study, leaving data from 77 participants to be analyzed (see Table 1 for sample characteristics). Participants were enrolled at the Center of Brain, Behavior and Metabolism in Lübeck, Germany between January 2019 and October 2022, with a break due to restrictions during the SARS-CoV-2 pandemic between March 2020 and October 2021 (see Supplementary Materials for complete list of exclusion and inclusion criteria and pandemic-related changes to the study protocol). An a priori power analysis with an alpha level of 0.05 and assuming an effect size f of 0.15 for a within-between-subject interaction had resulted in a required total sample size of *N* = 90 to achieve a power of 0.80. Due to the severe restrictions and delays caused by the SARS-CoV-2 pandemic, the target sample size was not fully achieved (*N* = 77 usable data sets). The actual power with this sample size was 0.74. Participants with AUD were recruited from addiction wards of two local inpatient psychiatric clinics. All had successfully completed physical withdrawal and had been abstinent for 9–39 days at the time of study participation (*M* = 14.3, median = 12, *SD* = 6.3 days). Control participants were recruited by public notices, online advertisements and by letter via the residents’ registration office. The control group was matched to the AUD group in terms of age and gender. This study was approved by the Ethics Committee of the University of Lübeck, Germany (AZ 17–077). Full written informed consent was obtained from all participants.

**Table 1:** Sample characteristics.

	AUD (*N* = 40)	Control group (*N* = 37)	Total (*N* = 77)	group comparison p-value
Gender				
female	2 (5.0%)	2 (5.4%)	4 (5.2%)	
male	38 (95.0%)	35 (94.6%)	73 (94.8%)	
Age [years]				
Mean (SD)	42.23 (9.15)	43.38 (9.68)	42.78 (9.36)	
Range	24–59	25–59	24–59	
Body mass index [kg/m^2^]				0.042
Mean (SD)	24.84 (3.56)	26.86 (4.95)	25.81 (4.38)	
Range	16.90–30.90	20.28–44.30	16.90–44.30	
Depressive symptoms [BDI-FS]				<0.001
N-Miss	0	1	1	
Mean (SD)	3.79 (2.97)	1.05 (1.33)	2.49 (2.71)	
Range	0–12	0–5	0–12	
Trait stress reactivity [SRS]				<0.001
Mean (SD)	56.32 (10.62)	47.22 (7.80)	51.95 (10.37)	
Range	36–81	36–67	36–81	
Chronic stress [TICS]				<0.001
Mean (SD)	21.17 (8.00)	10.68 (5.12)	16.13 (8.55)	
Range	2–36	3–24	2–36	

*p*-values derive from group comparisons using two-sample *t*-tests.

*BDI-FS* beck depression inventory fast screen [63], *SRS* stress-reactivity-scale [67], *TICS* screening scale of the trier inventory for the assessment of chronic stress [68].

### Study design and procedure

The present analysis was part of a larger functional magnetic resonance imaging (fMRI) study examining acute behavioral, physiological and neural responses to social stress in AUD. Findings on differential stress responses and associated functional connectivity from this sample have been published elsewhere [53]. After an initial screening process ensuring that all inclusion and no exclusion criteria were met, eligible participants were invited to two study visits. For all except for one control participant, these two visits were scheduled between 1 and 9 days apart (*M* = 2.59, *SD* = 2.01). For one control participant, the second visit had to be postponed due to indisposition, which resulted in it not taking place until 48 days after the first visit. Excluding this participant from the analyses did not affect the pattern of results.

**Fig. 1:**
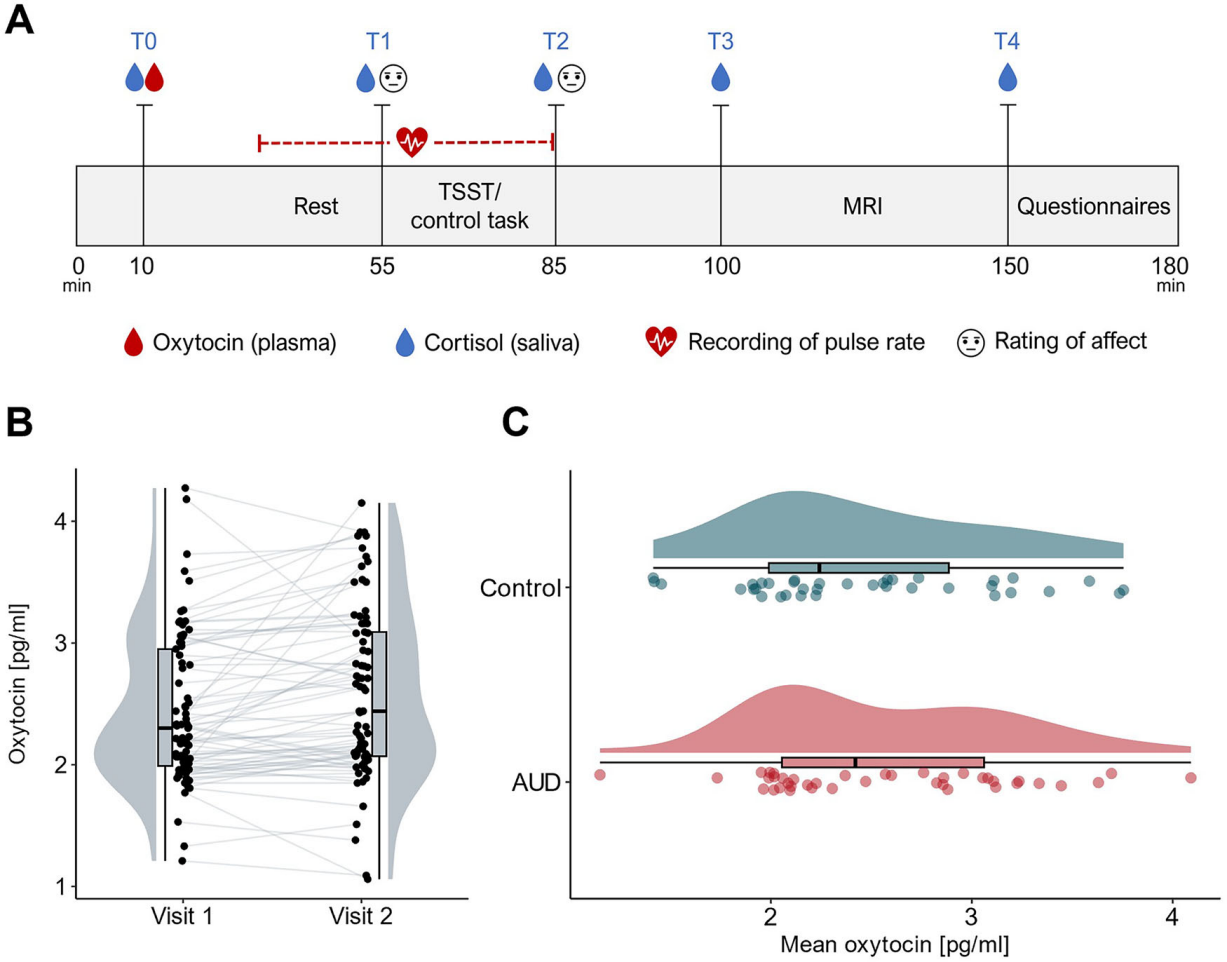
Overview of study design and oxytocin levels. **A** Course of the two study visits. The visits were identical in procedure except for an experimental manipulation of stress: participants either completed the Trier Social Stress Test (TSST) or a control task. **B** Within-subject variation of plasmatic oxytocin levels across two visits (jittered raw data from all participants, boxplots and probability distributions). **C** Averaged plasmatic oxytocin levels in participants with AUD and control participants (from top to bottom: probability distribution, boxplot, and jittered raw data for participants of each group).

Except for an experimental manipulation of social stress, the two visits were identical in procedure (Fig. 1A). The order of the stress and control day was counterbalanced across participants. At the beginning of each visit, it was ensured that all inclusion criteria were still met, which was then followed by the collection of a first saliva and blood sample. Subsequently, participants were introduced to the tasks they were to perform in the scanner and screened for recent MRI contraindications. After this, participants were told to rest while they were shown a video of landscape scenes for about 20–30 min. During this period, a pulse oximeter (PULOX PO-300, Novidion GmbH) was attached to the participant’s index finger of the non-dominant hand and pulse rate recordings were started. After the resting period, participants were asked to give a second saliva sample and rate their current level of anxiety using the state scale of the State-Trait Anxiety Inventory (STAI-s) [54]. They were then introduced to the stress or control task, which took 20 min to complete (for details, see below). After the task, pulse rate recordings were stopped and participants were again asked to provide a saliva sample and rate their level of anxiety. Another saliva sample was retrieved about 20 min later, which was immediately before participants entered the MRI (for details, see Supplementary Materials). Afterwards, another saliva sample was collected and participants filled out questionnaires assessing participant health and experienced chronic stress (for details, see below). Both visits lasted around 3 h in total and were conducted at the same time of day (8:30–11:30 a.m. ± 30 min.) to prevent day time fluctuations in the endocrinological parameters. Participants were advised to abstain from caffeine and exercise in the morning before the visits. Smoking was allowed up until 30 min before study participation.

### Stress and control task

The Trier Social Stress Test [55] was used to induce psychosocial stress. The TSST is a validated and widely used paradigm to assess the impact of stress under experimental conditions [56] and follows a standardized protocol, which consists of a 10 min anticipation phase and a 10 min test phase. By placing participants in a situation of social-evaluative threat, the TSST has been shown to produce reliable physiological stress responses such as increased heart rate and cortisol release [57]. Before the task started, participants were informed that their verbal and mathematical skills would be evaluated and that they would have to perform a short speech in front of a jury. In the anticipation phase, they had 10 min to prepare this speech, which they delivered in front of the jury in the subsequent test phase. Additionally, they were asked to perform a mental arithmetic task.

As a control condition, participants were asked to read a text aloud and solve easy arithmetic tasks in the absence of a jury, ostensibly to measure the physiological correlates of mental effort. During the anticipation phase of the control task, participants were instructed to read through the text. The TSST and the control task took place in the same rooms and participants were asked to stand during the test phase in both conditions. Thus, the control task was comparable to the TSST in terms of physical demands, but lacked the element of social evaluation.

### Cortisol and oxytocin samples

Salivary cortisol was measured from samples collected at five timepoints during the two visits (Fig. 1A). Saliva was collected using a cotton roll (Neutral Salivettes®, SARSTEDT, Nümbrecht, Germany) and stored in a freezer at −20 °C until it was sent to an external laboratory for analysis (Dresden LabService GmbH, Dresden, Germany). The samples were thawed and centrifuged at 3000 rpm for 5 min. Salivary cortisol levels were then determined using a chemiluminescence immunoassay procedure (IBL International GmbH, Hamburg, Germany). The intra- and inter-assay coefficients of variance were below 9%.

Baseline oxytocin levels were determined from blood samples taken at the beginning of each visit. The blood was collected into precooled 2.6 ml S-Monovettes® (SARSTEDT, Nümbrecht, Germany) and then centrifuged for 15 min at 4 °C and 1000 rpm. The obtained blood plasma was subsequently stored at −80 °C until the samples were analyzed by a third party (RIAgnosis, Sinzing, Germany) using a combination of selective extraction and extremely sensitive and specific radioimmunoassay (RIA). This standardized and validated procedure has frequently been used to quantify oxytocin in biological fluids including plasma, saliva, and cerebrospinal fluid (e.g., [20], [58], [59]) (for details, see Supplementary Materials). Intra- and inter-assay coefficients of variation were <10%.

### Questionnaires

State anxiety was measured using the state scale of the German short version of the State-Trait Anxiety Inventory (STAI-s) [54], which captures acute feelings of tension, apprehension, worry and nervousness. The STAI is a validated and widely used self-rating scale for measuring the severity of anxiety [60].

Additionally, all participants completed several self-report scales at the end of each visit, three of which were relevant to the present analysis. During the first visit, participants filled out the Beck Depression Inventory (BDI-II) [61, 62]. For the current analysis, we only included items capturing cognitive and affective symptoms, since it has been shown that using self-report measures of depression that include somatic complaints may lead to an overestimation of the prevalence of depression in patients with medical conditions, including substance use disorders (Volk et al., 1993). This procedure is analogous to using the Beck Depression Inventory Fast Screen for medical patients (BDI-FS) [63], which has been validated in various clinical populations [64–66].

At the end of the second visit, participants completed the Stress-Reactivity-Scale (SRS) [67], as well as the screening scale of the Trier Inventory for the Assessment of Chronic Stress (TICS) [68]. The SRS assesses a person’s tendency to respond to stressors with immediate, long lasting and intense stress reactions [67]. The TICS captures different aspects of subjectively experienced chronic stress, such as work overload or lack of social recognition, over the last three months [68].

### Statistical analysis

All statistical analyses were performed using R Statistical Software version 4.1.0 [69] (for details on used packages, see Supplementary Materials). For each participant and each visit, we calculated the difference in anxiety before and after the task, which was captured in the difference in STAI-s sum scores at T2 vs. T1. Pulse rate recordings were averaged across the resting period as well as the test phase of the stress or control task. Pulse rate change was quantified as the difference between mean pulse rate during the test phase and resting period, for each participant and visit. Cortisol change was calculated using the Area under the curve with respect to increase (AUCi) [70]. Salivary cortisol levels collected at T1 (after the resting period) were chosen as the reference point from which the increase was determined.

Following prior analyses [59], we estimated relative and absolute reliability of plasmatic oxytocin levels between the two visits using within-subject coefficients of variation (CV) and intraclass correlations (ICCs). ICCs across the two visits were calculated using a two-way mixed model, single measures, absolute agreement [71]. A two sample t-test was performed to compare mean oxytocin levels in participants with AUD and control participants.

The effects of oxytocin on affective and physiological markers of stress were assessed using linear mixed-effect models. Anxiety change, pulse rate change, as well as cortisol change were used as dependent variables. We constructed three models per dependent variable, each containing a random intercept for each participant, as well as task (TSST vs. control task), group (AUD vs. control group) and oxytocin as fixed effects. All models also controlled for gender, age, as well as the order of the stress vs. control task by including them as additional fixed effects. Age and oxytocin were centered on the grand mean. The models differed only in whether and which interaction effects were included: the first model only included main effects, the second model included all main effects and two-way interactions between oxytocin, task and group, and the third model included all main effects, two-way interactions and the three-way interaction of oxytocin × group × task. We used likelihood ratio tests to determine the model that best explained the data for each dependent variable (see Supplementary Table S4 for complete results of the model comparisons). Since the models only differed in their fixed effects structure, they were estimated using maximum likelihood (ML) for the purpose of model comparisons. This is recommended since likelihood values derived from restricted maximum likelihood (REML) depend on the fixed effects in the model [72]. The respective winning model was then re-estimated using REML and significance tests for the model coefficients were performed using Satterthwaite’s approximations for degrees of freedom [73].

To aid interpretation of non-significant results, Bayesian regression models were estimated using the brms package in R [74]. Bayesian analyses were conducted for the independent samples t-test comparing mean oxytocin levels between groups, as well as for the non-significant main and interaction effects in the linear mixed-effects models. For the t-test, default Cauchy priors were applied. For the linear mixed-effects models, normal priors were selected for the fixed effects, while remaining priors were adopted from the default_priors() function in the brms package.

## Results

### Good reliability of plasmatic oxytocin measures

Within-subject variation of plasmatic oxytocin measurements is illustrated in Fig. 1B. The ICC for the total sample was estimated to be 0.79, 95% CI [0.68; 0.87]. For the AUD group, the ICC was slightly lower at 0.71 [0.50; 0.84]. For the control group, it was 0.90 [0.81; 0.95]. The within-subject coefficient of variation (CV) was estimated to be 11.1% for the total sample, and 12.4% and 9.4% for the AUD and control groups, respectively. In general, the findings suggest good reliability of plasma oxytocin levels in our sample.

### No difference in mean oxytocin levels between groups

The distribution of oxytocin levels in the two groups is shown in Fig. 1C. Since oxytocin levels were relatively stable between the two visits, we used mean values when comparing oxytocin levels in participants with AUD (*M* = 2.56 pg/ml, *SD* = 0.63) and control participants (*M* = 2.46 pg/ml, *SD* = 0.63). Results of a two-sample t-test indicated that there were no significant differences between the groups (*t*(75) = 0.727, *p* = 0.470, *d* = 0.17 [−0.28; 0.61] with equal variances assumed, *p* = 0.81). This finding was supported by a Bayesian two-sample t-test, which provided moderate evidence for the null hypothesis over the alternative (Bayes Factor 10 (BF_10_) = 0.298).

### Oxytocin is associated with affective, but not physiological markers of stress

#### Oxytocin effects on anxiety change

**Fig. 2:**
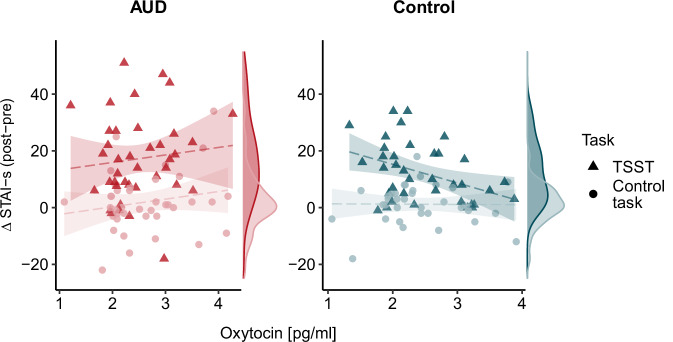
Relationship of oxytocin levels and anxiety change by task and group. Results of a linear mixed model showed a significant group × oxytocin interaction, indicating that mean oxytocin levels were differentially associated with anxiety change in the two groups across the two tasks. This interaction was mainly driven by changes during the TSST condition.

The model containing two-way interactions best explained the change in anxiety (R^2^ marginal = 0.32, R^2^ conditional = 0.42; see also Supplementary Tables S2, S3). A significant main effect of task suggested that anxiety change was higher in the TSST condition than the control condition (β = 6.96 [5.29, 8.64], *p* < 0.001). The main effect of oxytocin was not significant (β = 0.08 [−2.90, 3.07]). However, there was a significant oxytocin × group effect (β = 3.61 [0.62, 6.59]), *p* = 0.024), indicating that oxytocin levels were differentially associated with the overall anxiety change in participants with AUD and control participants irrespective of the task. To further explore the oxytocin × group interaction, we conducted post-hoc correlation analyses examining the relationship between mean oxytocin levels and mean change in anxiety separately for each group. The results showed that mean oxytocin levels were positively correlated with mean anxiety change in the AUD group (*r* = 0.274, *p* = 0.087) and negatively correlated in the control group (*r* = −0.274, *p* = 0.101), although both correlations did not reach statistical significance. A post-hoc power analysis for the test of the differences between the two correlations yielded a power of 0.76. Further correlation analyses revealed that this effect was mainly driven by differences in the TSST condition (Fig. 2), where the correlations of oxytocin and anxiety change differed significantly between groups (AUD: *r* = 0.223, controls: *r* = −0.407, *p* = 0.006, using Fisher’s r-to-Z transformation for the comparison of correlation coefficients). In contrast, the correlations of anxiety change and oxytocin did not differ between the groups under control conditions (AUD: *r* = 0.186, controls: *r* = −0.011, *p* = 0.403).

#### No oxytocin effects on pulse rate change

The model including two-way interactions best explained changes in pulse rate (R^2^ marginal = 0.12, R^2^ conditional = 0.58; see Supplementary Table S4). A significant main effect of task suggested a greater change in pulse rate in the TSST condition compared to the control condition (β = 2.58 [1.46, 3.73], *p* < 0.001). Further, there was a significant group × task interaction (β = −1.75 [−2.89, −0.62], *p* = 0.004), reflecting a less pronounced increase in pulse rate in the TSST condition (compared to the control condition) in AUD (see [53] for details). The main effect of oxytocin was not significant (β = −0.20 [−3.09, 2.68]) and there were no significant interactions of oxytocin with task (β = −1.01 [−2.79, 0.76]) or group (β = −1.16 [−4.08, 1.79]), indicating that oxytocin levels were not associated with the change of pulse rate over the course of the tasks. Bayesian model comparisons between the full model and reduced models, each excluding the respective oxytocin-related term, yielded Bayes factors of BF_10_ = 0.817 for the main effect of oxytocin, BF_10_ = 0.931 for the oxytocin × task interaction, and BF_10_ = 0.975 for the oxytocin × group interaction. These values provide anecdotal evidence in favor of the null hypothesis, suggesting that excluding the oxytocin terms did not meaningfully impair model fit.

#### No oxytocin effects on cortisol release

The model including two-way interactions best explained the increase in cortisol levels (R^2^ marginal = 0.21, R^2^ conditional = 0.43; see Supplementary Table S5). Again, a significant main effect of task suggested that the increase in cortisol levels was higher in the TSST condition than the control condition (β = 127.5 [86.0, 169.0], *p* < 0.001). The analysis also yielded a significant group × task interaction (β = −57.8 [−99.5, −16.2], *p* = 0.009), reflecting blunted cortisol responses to the stress intervention in AUD, as previously reported in the literature [7, 8]. The main effect of oxytocin was not significant (β = −32.4 [−115.7, 50.8]) and there were no significant interactions of oxytocin with task (β = −1.3 [−65.8, 63.7]) or group (β = 67.1 [−16.3, 150.6]). The results suggest that there was no relationship between plasmatic oxytocin levels and cortisol release over the course of the visits. Bayesian comparisons between the full model and the null models excluding the oxytocin-related term yielded Bayes factors of BF_10_ = 0.964 for the main effect of oxytocin, BF_10_ = 0.982 for the oxytocin × task interaction, and BF_10_ = 0.970 for the oxytocin × group interaction. These Bayes factors offer no evidence for either hypothesis and suggest that omitting these effects did not substantially alter model performance.

#### Exploratory analyses – altered association of oxytocin and depressive symptoms in AUD

**Fig. 3:**
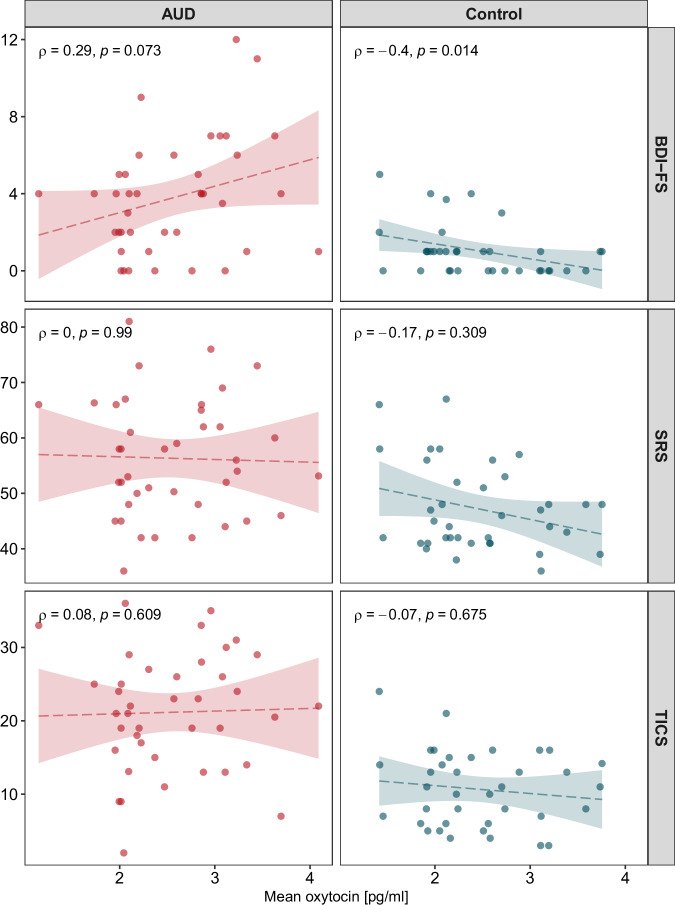
Correlations of mean oxytocin values with non-somatic symptoms of depression (BDI-FS), trait stress reactivity (SRS) and perceived chronic stress (TICS). The association between oxytocin levels and depressive symptoms was significantly different between the AUD and control groups, as indicated by a significant group × oxytocin interaction in a linear regression analysis.

Oxytocin levels seem to be associated with affective, but not physiological stress responses in our sample. To explore whether oxytocin levels were differentially associated with other measures of affective experiences, we performed three additional linear regressions examining the relationship between oxytocin and depressive symptoms, trait stress reactivity and perceived chronic stress. All models included mean oxytocin levels, group, as well as their interaction term as predictors. BDI-FS, SRS, as well as TICS screening scores were used as outcome variables, respectively. FDR correction on the main effects and interactions was applied to adjust for testing three models. There was a significant group × oxytocin interaction on BDI-FS scores (β = −2.15 [−3.81, −0.49], *p*_FDR_ = 0.036), suggesting that oxytocin levels were differentially associated with depressive symptoms in the two groups. Post-hoc correlation analyses showed that oxytocin levels were positively associated with depressive symptoms in the AUD group (Spearman ρ = 0.29, *p* = 0.073), and negatively associated with depressive symptoms in the control group (ρ = −0.40, *p* = 0.014; Fig. 3). Group, oxytocin or the interaction term did not significantly predict stress reactivity or perceived chronic stress (Supplementary Table S6).

## Discussion

In this study we investigated the relationship between baseline plasma oxytocin levels and responses to stress in individuals with AUD. Our main findings suggest an altered association of oxytocin and affective stress responses in individuals with AUD. While participants with AUD showed a positive relationship between plasmatic oxytocin levels and stress-induced anxiety change, the opposite relationship was found in control participants. A similar pattern of results was found for affective and cognitive symptoms of depression in exploratory analyses, indicating that higher oxytocin levels were associated with higher depression levels in individuals with AUD, but lower depression levels in control participants.

In healthy individuals, previous studies have shown that higher endogenous oxytocin levels correlate with reduced anxiety responses [27–29], a pattern that we also replicate in our control sample. However, in subjects with AUD, oxytocin appears not to have the same anxiolytic properties under stressful conditions. This finding might explain results from clinical intranasal oxytocin trials that were conducted expecting the same anxiolytic effects as in healthy participants, but showed diverging effects in subjects with depressive symptoms. For example, in a study examining oxytocin effects in individuals with depression, oxytocin led to increased anxiety levels over the course of a psychotherapy session, contrary to the authors’ hypothesis [50]. Furthermore, another study showed that in a subclinical sample participants with high depression scores who received oxytocin were unable to inhibit the processing of sad faces, which was also contrary to the authors’ expectation [75]. It is not clear what the mechanisms are why oxytocin might have a reduced anxiolytic or even anxiogenic effect in AUD. It is known that the stress axes are dysregulated in chronic alcohol consumption [6–12] which presumably affects the bidirectional interaction between oxytocin and stress systems [76]. The effects of oxytocin have also been shown to differ as a function of other individual differences such as coping style [77], or perceived social isolation [51]. It has been proposed that oxytocin modulates interoceptive signals and self-referential processing which can have a positive effect on some persons but a negative effect on others [52].

Assuming that affective responses to acute stress mediate addiction-related behaviors, this positive correlation of oxytocin levels and increased anxiety may suggest that individuals with AUD might not benefit from oxytocin administration. Indeed, recent findings from randomized controlled trials showed that intranasal oxytocin did not reduce alcohol craving [36, 37] or prevent relapse in patients with AUD after detoxification [36]. It is worth noting, however, that findings from clinical oxytocin trials have been heterogeneous, and studies on oxytocin effects in humans with AUD are still scarce [78]. Nevertheless, our results are in line with the notion that oxytocin effects are modulated by contextual and individual differences [79, 80] and highlight the importance of considering these differences when making predictions about the efficacy of oxytocin interventions.

While oxytocin levels were differentially correlated with changes in anxiety after stress in our sample, we found no significant relationship between oxytocin and pulse rate change or salivary cortisol increase in either group. At first glance, this finding seems to contradict evidence from animal and human studies showing that oxytocin administration has stress-buffering effects on the HPA-axis [18, 28, 81, 82] as well as the autonomic nervous system [83]. However, only few studies have examined associations of *endogenous* oxytocin and physiological stress responses, and the evidence from these studies has been heterogeneous. For example, higher overall plasmatic oxytocin levels were linked to a stronger decline in heart rate during a cold pressor task, but not during a public speaking task in postpartum mothers [24]. Another study showed that there was no correlation of oxytocin and cortisol increase in the TSST in older women [84]. Thus, our results align with previous findings on the relationship between plasma oxytocin levels and physiological changes triggered by psychosocial stress, which appear to differ from studies involving oxytocin administration.

We did not find evidence for elevated baseline oxytocin levels in patients with AUD compared to healthy control participants. It has been shown that oxytocin blood concentrations continuously decrease during the early stages of abstinence in individuals with AUD, with one study reporting no significant differences compared to a healthy control group after around five days [43]. Another study similarly reported a significant decrease of blood oxytocin levels after day 4, although oxytocin levels were still elevated compared to controls after one month of abstinence [44]. Since we included patients who had been abstinent for nine to 39 days at the time of participation, comparable oxytocin levels in AUD and control participants might result from this decline of oxytocin during the first time period of abstinence, although it still is unclear whether this decline takes place over only a few days or weeks. Together with previous evidence, this suggests that elevated blood oxytocin levels in AUD are a reversible consequence of excessive alcohol consumption, rather than a risk factor for addiction.

Our reliability analysis revealed that baseline plasmatic oxytocin measures were stable within individuals between the two visits. On a descriptive level, reliability measures tended to be lower within the AUD group, which might be explained by the aforementioned decline of oxytocin levels in the first weeks of abstinence [43, 44]. However, even within the AUD group, reliability measures were good and did not substantially differ from the control group. This finding stands in contrast to the only previous study that evaluated the reliability of peripheral oxytocin concentrations and observed that salivary and plasmatic oxytocin were not stable over time, concluding that single measurements are not valid trait markers of the oxytocin system in humans [59]. Importantly, our methods to quantify oxytocin concentrations and analyze their reliability were similar or even identical to those reported in Martins et al. [59]: (1) we collected blood samples at roughly the same time in the morning to avoid confounding effects of daytime fluctuations of oxytocin, (2) samples were processed immediately after their collection, (3) plasma samples were extracted prior to quantification, (4) quantification was carried out using radioimmunoassay in the same laboratory, thus following the same standard procedures, and (5) between-visits reliability was estimated using ICC and CV to ensure comparability of the results. While the cause of our diverging results is unclear, they do not support the conclusion that baseline plasma oxytocin levels are generally unstable under typical laboratory conditions. Since the samples of Martins et al. were obtained at the beginning of a pharmacological study in which the participants received oxytocin via different routes of intranasal administration, it could be that different examination procedures and associated anticipatory stress result in different intraindividual variability and thus reliability.

A strength of our study is that we were the first to empirically investigate associations of endogenous oxytocin system functioning and stress reactivity in AUD, which is an important step towards the understanding of addiction and the development of targeted pharmacological interventions. However, when interpreting the present results, one may consider the following aspects. First, the measurement of oxytocin concentrations is challenging. The radioimmunoassay used in the present study is considered a reliable method, but it is important to note that multiple valid methods may produce divergent results and a multitude of factors can influence oxytocin measurements [85, 86]. Second, plasma oxytocin, that is, peripheral oxytocin concentrations, were analyzed, although oxytocin is assumed to exert effects on stress responses mainly via central release [25]. While plasmatic oxytocin is regularly used in human studies since collecting blood samples is less invasive than collecting centrally circulating fluids, there have been doubts on whether peripheral oxytocin concentrations are a good approximation for central oxytocin concentrations. Research on the relationship of central and peripheral endogenous oxytocin has yielded mixed results (see [87] for review). A meta-analysis of animal and human studies found positive associations between central and peripheral oxytocin concentrations after experimentally induced stress and after intranasal oxytocin administration, but not under baseline conditions [88]. As long as there is not more clarity about these relationships and potential moderating variables that can explain the contradictory study results, it is difficult to draw conclusions with clinical implications. Third, oxytocin effects are often found to be moderated by interindividual differences, for example in early chronic stress experiences, bonding behavior, social support and variation in the oxytocin or oxytocin receptor gene [80], which we did not investigate here. Further, while we did include women in our study, our sample was predominantly male. Since there have been various reports of sex-dependent oxytocin effects on stress responses [15, 89], our conclusions may be limited to men only. Fourth, it is important to note that this was a correlational study, which means that our findings cannot be used to make claims about a causal role of oxytocin-system functioning in AUD. Whether altered associations of oxytocin and affective stress responses represent a risk factor for AUD or a consequence of prolonged alcohol consumption thus remains an open question. Fifth, the data collected with the pulse oximeter only allowed the pulse rate to be analyzed, but not the pulse rate variability, as the raw heart rate data is not available for the analyses. For future studies, it would be interesting to record heart rate variability, which provides more information about the functioning of the autonomic nervous system.

To conclude, our findings suggest that endogenous oxytocin levels may not be altered at baseline in individuals with AUD during abstinence, but rather are differentially related to affective responses to psychosocial stress. This indicates that the effects of oxytocin are complex and depend on individual factors that have not yet been sufficiently addressed. Although no oxytocin administration took place in this study, the results could indicate that administering exogenous oxytocin might not help individuals with AUD – at least during the early stages of abstinence – if affective responses to stress drive addiction-related behaviors like craving and relapse. While it is likely that elevated baseline oxytocin levels during the first days of abstinence are a reversible consequence of prolonged alcohol consumption [43], future studies should address whether altered associations of oxytocin and stress-related anxiety persist even in long-term abstinence.

## Supplementary information

Supplemental material

## Data Availability

Data and code of this study are openly available at: https://osf.io/gjs5h/.
