# Supplementary material for: Altered association of plasmatic oxytocin with affective stress response in alcohol use disorder
Source: Transl Psychiatry. 2025 Oct 20;15:421. doi: 10.1038/s41398-025-03522-0 (PMC12537864; doi:10.1038/s41398-025-03522-0)
Supplement: Supplementary file 1 — Supplemental material [file 41398_2025_3522_MOESM1_ESM.docx]

Supplementary Materials for: Altered association of plasmatic oxytocin with affective stress responses in alcohol use disorder

Mayer, A.V., Schwarze, Y., Stierand, J., Voges, J., Schröder, A., von der Gablentz, J., Junghanns, K., Voß, O., Krach, S., Paulus, F.M., & Rademacher, L.

**Contents**

[**Inclusion and exclusion criteria 1**](#_8v5kyok2no3)

[**Changes in study protocol due to SARS-CoV-2 pandemic 1**](#_nreiqil3dw3w)

[**MRI experiments 2**](#_916io1ple01v)

[**Quantification of oxytocin in plasma samples 2**](#_bhnm1s69p03q)

[**Analysis software 3**](#_p2qrwvcicaja)

[**Supplementary Tables 4**](#_kr7p9llsr39o)

[Table S1. Summary of affective and physiological stress measures per task and group. 4](#_dttrij4vcwwg)

[Table S2: Model comparisons of linear mixed-effect models. 5](#_3s8p6z7ca3qr)

[Table S3: Oxytocin and anxiety change: results of linear mixed-effects model. 6](#_7xndfa9zybbl)

[Table S4: Oxytocin and pulse rate change: results of linear mixed-effects model. 7](#_dz3l5bxk14je)

[Table S5: Oxytocin and cortisol increase: results of linear mixed-effects model. 8](#_62jykn8nq065)

[Table S6: Oxytocin, stress and depression: results of linear regression analyses. 9](#_cy254vgdqer5)

[**References 10**](#_209jcz97b6yo)

#

#

# Inclusion and exclusion criteria

General inclusion criteria were an age between 18 and 60 years and sufficient

German language skills. Participants were excluded if they (1) suffered from any neurological disease, traumatic brain injuries, acute inflammatory disease or chronic inflammatory bowel disease, (2) were taking medication that exerted a direct influence on the HPA axis, in particular beta-blockers or medication containing hydrocortisone. Since parts of this study were carried out using magnetic resonance imaging (MRI), subjects were also required to be free of any metallic implants such as pacemakers, defibrillators or copper IUDs, as well as metallic splinters or shrapnel. Other MRI contraindications included large tattoos or permanent make-up, claustrophobia, and pregnancy.

Participants with alcohol use disorder (AUD) were additionally required to meet the criteria for moderate to severe AUD according to the Diagnostic and Statistical Manual of Mental Disorders fifth edition (DSM-5) and to be abstinent for at least 9 days at the time of study participation. Participants with AUD were excluded if they (1) had a history of substance abuse or dependency other than alcohol or nicotine within the last 12 months, (2) were diagnosed with major depression, social anxiety disorder, antisocial or borderline personality disorder, (3) were in an acute state of psychosis or suicidality. Control participants were excluded if they (1) suffered from any current or past psychiatric disorders, (2) scored ≥8 on the Alcohol Use Disorder Identification Test (AUDIT) [^1^](https://www.zotero.org/google-docs/?k9axc0), (3) had any first-degree relatives with alcohol use disorder, (4) had consumed drugs (except for alcohol, nicotine, and irregular use of cannabis) in the past year. All inclusion and exclusion criteria were checked by telephone or in a personal interview before participants were included in the study.

# Changes in study protocol due to SARS-CoV-2 pandemic

Due to the SARS-CoV-2 pandemic, data collection was temporarily halted between March 2020 and October 2021. The study was continued as soon as the risk for infection was sufficiently reduced by available vaccinations and testing. Forty-nine participants were enrolled before the pandemic, and thirty-one participants were enrolled during the pandemic. Following the pause, several procedural adjustments were made. For instance, participants underwent a rapid COVID-19 test upon arrival, and face masks were worn throughout the visits. Additionally, before the pandemic, blood samples were collected at four time points using indwelling venous catheters: ten minutes after arrival (T0), immediately after the stress or control task (T2), after MRI measurements (T4) and at the end of each visit (T5). During the pandemic, we decided to reduce the number of blood draws. Blood was now only collected at the beginning (T0) and end (T5) of each study visit using venipuncture.

# MRI experiments

The present analysis is part of a larger fMRI study investigating the acute behavioral, physiological, and neural responses to social stress in AUD, involving two MRI experiments for participants. Both experiments examined brain function associated with social vs. non-social stimuli, which might be altered after stress in individuals with AUD. The first experiment was a modified version of the monetary and social incentive delay (MID/SID) paradigm [^2–4^](https://www.zotero.org/google-docs/?0IGVia), which is a well-established task to study participants' neural responses to anticipated rewards. During the task, participants respond as quickly as possible to a target stimulus after visual cues indicate a potential reward, with fast responses earning either social rewards (smiling faces) or monetary rewards (small amounts of money). This task is known to activate the dopaminergic reward system, which has been associated with addiction and stress. The second experiment involved a novel paradigm aimed at exploring neural and behavioral responses to images of social situations. Participants viewed images of both social and non-social scenes with differing numbers of people and were then asked to rate how much they would like to be in the depicted situation. Results of the fMRI analyses will be reported elsewhere.

# Quantification of oxytocin in plasma samples

Blood plasma samples were analyzed by a third party (RIAgnosis, Sinzing, Germany) using a combination of selective extraction and extremely sensitive and specific radioimmunoassay (RIA). In brief, plasma samples (0.5ml) were kept at -20°C until extraction using LiChroprep® Si60 (Merck) heat-activated at 690°C for 3h. 20mg of LiChroprep® Si60 in 1ml distilled water were added to the sample, mixed for 30min, washed twice with distilled water and 0.01mol/l HCl and eluded with 60% acetone. To each evaporate, 50µl of assay buffer was added followed by 50µl rabbit antibody against oxytocin. After a 60-min preincubation interval, 10µl 125I-labeled tracer (PerkinElmer, Waltham, MA, USA) was added and samples incubated for three days at 4ºC. Unbound radioactivity was precipitated by activated charcoal (Sigma-Aldrich, St Louis, MO, USA). Under these conditions, an average of 50% of total counts are bound with <5% non-specific binding. The detection limit of the radioimmunoassay was in the 0.1-0.5 pg/sample range, depending on the age of the tracer, with typical displacements of 20–25% at 2 pg, 60–70% at 8 pg, and 90% at 32 pg of standard neuropeptide. Cross-reactivities with arginine vasopressin, ring moieties and terminal tripeptides of both oxytocin and vasopressin, as well as a wide variety of peptides comprising 3 (alpha-melanocyte-stimulating hormone) up to 41 (corticotropin-releasing factor) amino acids are <0.7% throughout. Serial dilutions of plasma samples containing high levels of endogenous oxytocin run strictly parallel to the standard curve indicating immuno-identity.

# Analysis software

We used R version 4.3.3 ^[5](https://www.zotero.org/google-docs/?d2fPiv)^ and the following R packages: afex v. 1.3.0 ^[6](https://www.zotero.org/google-docs/?yzA0Ws)^, arsenal v. 3.6.3 ^[7](https://www.zotero.org/google-docs/?58a6Mj)^, cocor v. 1.1.3 ^[8](https://www.zotero.org/google-docs/?xZr0Xn)^, DescTools v. 0.99.48 ^[9](https://www.zotero.org/google-docs/?9lFvoR)^, effectsize v. 0.8.5 ^[10](https://www.zotero.org/google-docs/?g7mdXj)^, ggExtra v. 0.10.1 ^[11](https://www.zotero.org/google-docs/?kRRdIf)^, ggpubr v. 0.6.0 ^[12](https://www.zotero.org/google-docs/?LyFvn5)^, ggrain v. 0.0.3 ^[13](https://www.zotero.org/google-docs/?lExpFW)^, irr v. 0.84.1 ^[14](https://www.zotero.org/google-docs/?d0tm6k)^, lme4 v. 1.1.28 ^[15](https://www.zotero.org/google-docs/?pKQDYz)^, mlmtools v. 1.0.2 ^[16](https://www.zotero.org/google-docs/?Mp9ld9)^, sjPlot v. 2.8.16 ^[17](https://www.zotero.org/google-docs/?xKmJJk)^, and brms v. 2.22.0 ^18^.

# Supplementary Tables

## Table S1. Summary of affective and physiological stress measures per task and group.

|  | **AUD group (N=40)** | | **Control group (N=37)** | |
| --- | --- | --- | --- | --- |
|  | **Control task** | **TSST** | **Control task** | **TSST** |
| **Anxiety pre task [STAI-S]** |  |  |  |  |
| Mean (SD) | 24.34 (10.12) | 23.15 (9.96) | 23.57 (8.27) | 22.49 (8.40) |
| Range | 12 - 57 | 10 - 52 | 10 - 41 | 10 - 46 |
| **Anxiety post task [STAI-S]** |  |  |  |  |
| Mean (SD) | 26.28 (10.39) | 41.48 (13.89) | 24.76 (7.69) | 34.70 (12.28) |
| Range | 12 - 50 | 12 - 73 | 10 - 39 | 10 - 59 |
| **Cortisol T0 [nmol/l]** |  |  |  |  |
| Mean (SD) | 9.22 (5.79) | 8.98 (5.43) | 8.22 (4.89) | 8.68 (5.49) |
| Range | 2.38 - 23.39 | 2.21 - 23.17 | 1.74 - 27.39 | 0.99 - 24.28 |
| **Cortisol T1 [nmol/l]** |  |  |  |  |
| Mean (SD) | 6.76 (5.97) | 5.95 (4.85) | 8.35 (7.20) | 8.64 (8.72) |
| Range | 0.95 - 38.50 | 1.35 - 29.75 | 1.54 - 34.14 | 1.28 - 37.83 |
| **Cortisol T2 [nmol/l]** |  |  |  |  |
| Mean (SD) | 5.01 (3.74) | 5.36 (3.34) | 6.49 (5.84) | 10.20 (7.52) |
| Range | 1.02 - 23.53 | 1.30 - 18.80 | 1.23 - 23.68 | 1.82 - 27.80 |
| **Cortisol T3 [nmol/l]** |  |  |  |  |
| N-Miss | 1 | 0 | 0 | 0 |
| Mean (SD) | 4.05 (2.63) | 6.19 (3.29) | 5.24 (3.57) | 13.28 (8.40) |
| Range | 1.13 - 13.94 | 1.47 - 14.81 | 1.12 - 13.70 | 1.63 - 32.54 |
| **Cortisol T4 [nmol/l]** |  |  |  |  |
| Mean (SD) | 4.19 (4.20) | 4.26 (2.74) | 5.00 (5.20) | 6.52 (3.95) |
| Range | 1.35 - 28.05 | 0.83 - 17.20 | 0.95 - 32.85 | 1.07 - 19.59 |
| **Cortisol increase from T1 [AUCi]** |  |  |  |  |
| N-Miss | 1 | 0 | 0 | 0 |
| Mean (SD) | 165 (219) | 300 (216) | 155 (220) | 524 (494) |
| Range | -417 - 956 | -92 - 1001 | -767- 636 | -366 - 2027 |
| **Pulse rate pre task [bpm]** |  |  |  |  |
| N-Miss | 0 | 0 | 1 | 0 |
| Mean (SD) | 81.29 (12.13) | 79.82 (13.18) | 71.16 (11.70) | 71.91 (12.52) |
| Range | 56.12 - 111.03 | 51.19 - 104.43 | 48.76 - 100.13 | 48.43 - 105.80 |
| **Pulse rate during task [bpm]** |  |  |  |  |
| N-Miss | 0 | 0 | 1 | 1 |
| Mean (SD) | 92.34 (13.44) | 92.59 (19.07) | 79.64 (12.20) | 89.98 (17.75) |
| Range | 69.94 - 124.79 | 54.99 - 130.30 | 55.42 - 103.92 | 53.22 - 135.15 |
| *Note.* STAI-S: State-Trait Anxiety Inventory - State measure ^[1](https://www.zotero.org/google-docs/?hzXTwb)9^. Higher STAI-S values indicate higher levels of subjectively perceived nervousness, tension, and worry. AUCi: area under the curve with respect to increase ^20^, calculated between T1 and T4. Higher AUCi indicates a stronger increase in cortisol levels between T1 and T4. Negative values indicate a decrease of cortisol levels between T1 and T4. | | | | |

## Table S2: Model comparisons of linear mixed-effect models.

| **DV** | **Model name** | **Model fit** | | | **LRT against nested** | | |
| --- | --- | --- | --- | --- | --- | --- | --- |
|  |  | **AIC** | **BIC** | **LL** | **df** | **χ^2^** | ***p*** |
| Anxiety change | main effects model | 1202.9 | 1230.2 | -592.4 |  |  |  |
|  | **two-way interactions model** | 1200.4 | 1236.8 | -588.2 | 3 | 8.51 | 0.037 |
|  | three-way interaction model | 1199.6 | 1239.1 | -586.8 | 1 | 2.81 | 0.094 |
|  |  |  |  |  |  |  |  |
| Pulse rate change | main effects model | 1135.5 | 1162.7 | -558.8 |  |  |  |
|  | **two-way interactions model** | 1130.7 | 1167.0 | -553.4 | 3 | 10.79 | 0.013 |
|  | three-way interaction model | 1132.7 | 1172.0 | -553.4 | 1 | 0.00 | 0.994 |
|  |  |  |  |  |  |  |  |
| Cortisol change | main effects model | 2200.1 | 2227.4 | -1091.1 |  |  |  |
|  | **two-way interactions model** | 2196.1 | 2232.4 | -1086.0 | 3 | 10.09 | 0.018 |
|  | three-way interaction model | 2197.9 | 2237.3 | -1085.9 | 1 | 0.19 | 0.664 |
| *Note*. We constructed three models per dependent variable, each containing a random intercept for each participant, as well as task (TSST vs. control task), group (AUD vs. control group) and oxytocin as fixed effects. The models differed only in whether and which interaction effects were included: the first model only included main effects, the second model included all main effects and two-way interactions between oxytocin, task and group, and the third model included all main effects, two-way interactions and the three-way interaction of oxytocin × group × task. All models were estimated using maximum likelihood (ML) for the purpose of model comparisons. The respective winning models are highlighted in bold. DV: dependent variable, LRT = likelihood ratio test, AIC = Akaike information criterion, BIC = Bayesian information criterion, LL = log likelihood. | | | | | | | |

## Table S3: Oxytocin and anxiety change: results of linear mixed-effects model.

| **Fixed effects** | | | | | | |
| --- | --- | --- | --- | --- | --- | --- |
|  | Beta | SE | 95% CI | | *t* | *p* |
|  |  |  | lower | upper |  |  |
| Intercept | 5.77 | 2.24 | 1.52 | 10.01 | 2.57 | 0.012 |
| Group | 1.71 | 0.99 | -0.18 | 3.59 | 1.72 | 0.090 |
| Age | 0.07 | 0.12 | -0.15 | 0.29 | 0.57 | 0.571 |
| Gender | -2.75 | 2.24 | -6.99 | 1.49 | -1.23 | 0.223 |
| Task | 6.96 | 0.86 | 5.29 | 8.64 | 8.10 | **< 0.001** |
| Order | 1.23 | 1.02 | -0.71 | 3.16 | 1.20 | 0.233 |
| Oxytocin | 0.08 | 1.57 | -2.90 | 3.07 | 0.05 | 0.959 |
| Group × Oxytocin | 3.61 | 1.57 | 0.62 | 6.59 | 2.29 | **0.024** |
| Group × Task | 1.46 | 0.86 | -0.22 | 3.13 | 1.69 | 0.095 |
| Task × Oxytocin | -0.71 | 1.34 | -3.25 | 2.01 | -0.53 | 0.595 |
|  |  |  |  |  |  |  |
| **Random effects** | | | | | | |
|  | Variance |  | SD |  |  |  |
| Participant (Intercept) | 18.2 |  | 4.3 |  |  |  |
|  |  |  |  |  |  |  |
| **Model fit** | | | | | | |
| R^2^ | Marginal |  | Conditional |  |  |  |
|  | 0.32 |  | 0.42 |  |  |  |
| *Note.* P-values for fixed effects have been calculated using Satterthwaite's approximations.  Model equation: anxiety difference ~ (1\|id)+ group + age + gender + task + order + oxytocin + oxytocin*group + group*task + oxytocin*task | | | | | | |

## Table S4: Oxytocin and pulse rate change: results of linear mixed-effects model.

| **Fixed effects** | | | | | | |
| --- | --- | --- | --- | --- | --- | --- |
|  | Beta | SE | 95% CI | | *t* | *p* |
|  |  |  | lower | upper |  |  |
| Intercept | 12.97 | 2.41 | 8.39 | 17.55 | 5.38 | <0.001 |
| Group | -0.81 | 1.04 | -2.79 | 1.16 | -0.78 | 0.438 |
| Age | -0.11 | 0.12 | -0.34 | 0.11 | -0.95 | 0.347 |
| Gender | 0.33 | 2.41 | -4.25 | 4.91 | 0.14 | 0.891 |
| Task | 2.58 | 0.58 | 1.46 | 3.73 | 4.44 | **< 0.001** |
| Order | -1.49 | 1.06 | -3.51 | 0.52 | -1.41 | 0.164 |
| Oxytocin | -0.20 | 1.52 | -3.09 | 2.68 | -0.13 | 0.895 |
| Group × Oxytocin | -1.16 | 1.52 | -4.08 | 1.79 | -0.76 | 0.447 |
| Group × Task | -1.75 | 0.58 | -2.89 | -0.62 | -2.99 | **0.004** |
| Task × Oxytocin | -1.01 | 0.92 | -2.79 | 0.76 | -1.10 | 0.274 |
|  |  |  |  |  |  |  |
| **Random effects** | | | | | | |
|  | Variance |  | SD |  |  |  |
| Participant (Intercept) | 56.39 |  | 7.51 |  |  |  |
|  |  |  |  |  |  |  |
| **Model fit** | | | | | | |
| R^2^ | Marginal |  | Conditional |  |  |  |
|  | 0.12 |  | 0.58 |  |  |  |
| Note. *P*-values for fixed effects have been calculated using Satterthwaite's approximations.  Model equation: pulse rate difference ~ (1\|id)+ group + age + gender + task + order + oxytocin + oxytocin*group + group*task + oxytocin*task | | | | | | |

## Table S5: Oxytocin and cortisol increase: results of linear mixed-effects model.

| **Fixed effects** | | | | | | |
| --- | --- | --- | --- | --- | --- | --- |
|  | Beta | SE | 95% CI | | *t* | *p* |
|  |  |  | lower | upper |  |  |
| Intercept | 208.2 | 63.9 | 87.1 | 329.3 | 3.26 | 0.002 |
| Group | -52.5 | 28.4 | -106.4 | 1.3 | -1.85 | 0.069 |
| Age | -0.8 | 3.3 | -7.0 | 5.5 | -0.23 | 0.818 |
| Gender | -82.7 | 63.8 | -203.6 | 38.2 | -1.30 | 0.199 |
| Task | 127.5 | 21.4 | 86.0 | 169.0 | 5.97 | **< 0.001** |
| Order | 8.0 | 29.1 | -47.1 | 63.1 | 0.28 | 0.784 |
| Oxytocin | -32.4 | 43.9 | -115.7 | 50.8 | -0.74 | 0.461 |
| Group × Oxytocin | 67.1 | 44.0 | -16.3 | 150.6 | 1.53 | 0.130 |
| Group × Task | -57.8 | 21.4 | -99.5 | -16.2 | -2.70 | **0.009** |
| Task × Oxytocin | -1.3 | 33.5 | -65.8 | 63.7 | -0.04 | 0.968 |
|  |  |  |  |  |  |  |
| **Random effects** | | | | | | |
|  | Variance |  | SD |  |  |  |
| Participant (Intercept) | 26017 |  | 161 |  |  |  |
|  |  |  |  |  |  |  |
| **Model fit** | | | | | | |
| R^2^ | Marginal |  | Conditional |  |  |  |
|  | 0.21 |  | 0.43 |  |  |  |
| Note. *P*-values for fixed effects have been calculated using Satterthwaite's approximations.  Model equation: AUCi ~ (1\|id)+ group + age + gender + task + order + oxytocin + oxytocin*group + group*task + oxytocin*task | | | | | | |

## Table S6: Oxytocin, stress and depression: results of linear regression analyses.

|  | **Depression [BDI-FS]** | | | |  | **Stress reactivity [SRS]** | | | |  | | **Chronic stress [TICS]** | | |  |
| --- | --- | --- | --- | --- | --- | --- | --- | --- | --- | --- | --- | --- | --- | --- | --- |
| Predictors | β | *CI* | *p* | *p_FDR_* | | β | *CI* | *p* | *p_FDR_* | | β | | *CI* | *p* | *p_FDR_* |
| Intercept | 0.28 | -2.74 – 3.30 | 0.854 |  | | 57.55 | 45.07 – 70.04 | **<0.001** |  | | 20.24 | | 11.11 – 29.37 | **<0.001** |  |
| Mean oxytocin | 1.37 | 0.22 – 2.52 | **0.020** | 0.060 | | -0.48 | -5.22 – 4.26 | 0.840 | 0.840 | | 0.36 | | -3.10 – 3.83 | 0.836 | 0.840 |
| Group | 2.68 | -1.59 – 6.96 | 0.215 | 0.435 | | -1.68 | -19.37 – 16.01 | 0.850 | 0.850 | | -6.92 | | -19.86 – 6.02 | 0.290 | 0.435 |
| Mean oxytocin × Group | -2.15 | -3.81 – -0.49 | **0.012** | **0.036** | | -3.04 | -9.89 – 3.81 | 0.379 | 0.569 | | -1.44 | | -6.45 – 3.58 | 0.570 | 0.570 |
| Observations | 76 | | | |  | 77 | | | |  | | 77 | | |  |
| R^2^ / R^2^ adjusted | 0.328 / 0.300 | | | |  | 0.217 / 0.185 | | | |  | | 0.384 / 0.358 | | |  |
| *Note.* BDI-FS: Beck Depression Inventory Fast Screen ^[21](https://www.zotero.org/google-docs/?VdUNTx)^, SRS: Stress-Reactivity-Scale ^[22](https://www.zotero.org/google-docs/?c3s8wp)^, TICS: screening scale of the Trier Inventory for the Assessment of Chronic Stress ^[23](https://www.zotero.org/google-docs/?bQbJZe)^. Significant effects are highlighted in bold. *p_FDR_* refers to p-values after FDR correction of the main effects and interactions for testing the three different dependent variables. | | | | | | | | | | | | | | |  |

# References

[1 Bohn MJ, Babor TF, Kranzler HR. The Alcohol Use Disorders Identification Test (AUDIT): validation of a screening instrument for use in medical settings.](https://www.zotero.org/google-docs/?sKQWLY) *[J Stud Alcohol](https://www.zotero.org/google-docs/?sKQWLY)* [1995;](https://www.zotero.org/google-docs/?sKQWLY) **[56](https://www.zotero.org/google-docs/?sKQWLY)**[: 423–432.](https://www.zotero.org/google-docs/?sKQWLY)

[2 Knutson B, Adams CM, Fong GW, Hommer D. Anticipation of increasing monetary reward selectively recruits nucleus accumbens.](https://www.zotero.org/google-docs/?sKQWLY) *[J Neurosci](https://www.zotero.org/google-docs/?sKQWLY)* [2001; **21**: RC159–RC159.](https://www.zotero.org/google-docs/?sKQWLY)

[3 Rademacher L, Krach S, Kohls G, Irmak A, Gründer G, Spreckelmeyer KN. Dissociation of neural networks for anticipation and consumption of monetary and social rewards.](https://www.zotero.org/google-docs/?sKQWLY) *[NeuroImage](https://www.zotero.org/google-docs/?sKQWLY)* [2010; **49**: 3276–3285.](https://www.zotero.org/google-docs/?sKQWLY)

[4 Spreckelmeyer KN, Krach S, Kohls G, Rademacher L, Irmak A, Konrad K *et al.* Anticipation of monetary and social reward differently activates mesolimbic brain structures in men and women.](https://www.zotero.org/google-docs/?sKQWLY) *[Soc Cogn Affect Neurosci](https://www.zotero.org/google-docs/?sKQWLY)* [2009;](https://www.zotero.org/google-docs/?sKQWLY) **[4](https://www.zotero.org/google-docs/?sKQWLY)**[: 158–165.](https://www.zotero.org/google-docs/?sKQWLY)

[5 R Core Team.](https://www.zotero.org/google-docs/?sKQWLY) *[R: A Language and Environment for Statistical Computing](https://www.zotero.org/google-docs/?sKQWLY)*[. R Foundation for Statistical Computing: Vienna, Austria, 2021. https://www.R-project.org/.](https://www.zotero.org/google-docs/?sKQWLY)

[6 Singmann H, Bolker B, Westfall J, Aust F, Ben-Shachar MS. *afex: Analysis of Factorial Experiments*. 2023https://CRAN.R-project.org/package=afex.](https://www.zotero.org/google-docs/?sKQWLY)

[7 Heinzen E, Sinnwell J, Atkinson E, Gunderson T, Dougherty G. *arsenal: An Arsenal of `R’ Functions for Large-Scale Statistical Summaries*. 2021https://CRAN.R-project.org/package=arsenal.](https://www.zotero.org/google-docs/?sKQWLY)

[8 Diedenhofen B, Musch J. cocor: A Comprehensive Solution for the Statistical Comparison of Correlations.](https://www.zotero.org/google-docs/?sKQWLY) *[PLOS ONE](https://www.zotero.org/google-docs/?sKQWLY)* [2015;](https://www.zotero.org/google-docs/?sKQWLY) **[10](https://www.zotero.org/google-docs/?sKQWLY)**[: e0121945–e0121945.](https://www.zotero.org/google-docs/?sKQWLY)

[9 Signorell A.](https://www.zotero.org/google-docs/?sKQWLY) *[DescTools: Tools for Descriptive Statistics](https://www.zotero.org/google-docs/?sKQWLY)*[. 2023https://CRAN.R-project.org/package=DescTools.](https://www.zotero.org/google-docs/?sKQWLY)

[10 Ben-Shachar MS, Lüdecke D, Makowski D. Effectsize: Estimation of Effect Size Indices and Standardized Parameters. *J Open Source Softw* 2020; **5**: 2815.](https://www.zotero.org/google-docs/?sKQWLY)

[11 Attali D, Baker C. *ggExtra: Add Marginal Histograms to `ggplot2’, and More `ggplot2’ Enhancements*. 2023https://CRAN.R-project.org/package=ggExtra.](https://www.zotero.org/google-docs/?sKQWLY)

[12 Kassambara A. *ggpubr: `ggplot2’ Based Publication Ready Plots*. 2023https://CRAN.R-project.org/package=ggpubr.](https://www.zotero.org/google-docs/?sKQWLY)

[13 Allen M, Poggiali D, Whitaker K, Marshall TR, Langen J van, Kievit RA. Raincloud plots: a multi-platform tool for robust data visualization [version 2; peer review: 2 approved]. *Wellcome Open Res* 2021; **4**. doi:10.12688/wellcomeopenres.15191.2.](https://www.zotero.org/google-docs/?sKQWLY)

[14 Gamer M, Lemon J, Singh P. *irr: Various Coefficients of Interrater Reliability and Agreement*. 2019https://CRAN.R-project.org/package=irr.](https://www.zotero.org/google-docs/?sKQWLY)

[15 Bates D, Mächler M, Bolker B, Walker S. Fitting Linear Mixed-Effects Models Using lme4. *J Stat Softw* 2015; **67**: 1–48.](https://www.zotero.org/google-docs/?sKQWLY)

[16 Jamison L, Mazen J, Ruzek E. *mlmtools: Multi-Level Model Assessment Kit*. 2022https://CRAN.R-project.org/package=mlmtools.](https://www.zotero.org/google-docs/?sKQWLY)

[17 Lüdecke D. *sjPlot: Data Visualization for Statistics in Social Science*. 2024https://](https://www.zotero.org/google-docs/?sKQWLY)[CRAN.R-project.org/package=sjPlot](http://cran.r-project.org/package=sjPlot)[.](https://www.zotero.org/google-docs/?sKQWLY)

18 Bürkner PC. Brms. An R package for Bayesian multilevel models using Stan. *J Stat Softw* 2017; 80: 1–28.

[19 Laux L, Glanzmann P, Schaffner P, Spielberger CD. *Das State-Trait-Angstinventar (STAI) : theoretische Grundlagen und Handanweisung*. Beltz: Weinheim, 1981.](https://www.zotero.org/google-docs/?sKQWLY)

20 [Pruessner JC, Kirschbaum C, Meinlschmid G, Hellhammer DH. Two formulas for computation of the area under the curve represent measures of total hormone concentration versus time-dependent change. *Psychoneuroendocrinology* 2003; **28**: 916–931.](https://www.zotero.org/google-docs/?sKQWLY)

[21 Beck AT, Steer R, Brown G. BDI-Fast Screen for Medical Patients Manual. Psychological Corporation. 2000.](https://www.zotero.org/google-docs/?sKQWLY)

[22 Schulz P, Jansen LJ, Schlotz W. Stressreaktivität: Theoretisches Konzept und Messung. *Diagnostica* 2005; **51**: 124–133.](https://www.zotero.org/google-docs/?sKQWLY)

[23 Schulz P, Schlotz W. Trierer Inventar zur Erfassung von chronischem Streß (TICS): Skalenkonstruktion, teststatistische Überprüfung und Validierung der Skala Arbeitsüberlastung. *Diagnostica* 1999; **45**: 8–19.](https://www.zotero.org/google-docs/?sKQWLY)
